# Supplementary material for: Rapid turnover of life-cycle-related genes in the brown algae
Source: Genome Biol. 2019 Feb 14;20:35. doi: 10.1186/s13059-019-1630-6 (PMC6374913; doi:10.1186/s13059-019-1630-6)
Supplement: Supplementary file 3 — Protein alignments, corresponding Gblocks html file and CDS sequences for the lineage conserved gametophyte-biased genes. (ZIP 2109 kb) [file 13059_2019_1630_MOESM3_ESM.zip › Con_GA_alignments/Ec-19_004670.aa_ali.fasta-gb.htm]

Ec-19\_004670.aa.fasta.trans.aa\_ali.fasta


## Gblocks 0.91b Results

Processed file: **Ec-19\_004670.aa.fasta.trans.aa\_ali.fasta**  
Number of sequences: **2**  
Alignment assumed to be: **Protein**  
New number of positions: **469** (selected positions are underlined in blue)

```
                         10        20        30        40        50        60
                 =========+=========+=========+=========+=========+=========+
Ec-19_004670     ------------------------------------------------------------
MSTRG.47812.1    XAVGQARDTEMERQEGHLKSEELEAKSSIERRDAKSPRDKRETSTADDKTAATGGGEDGG
                                                                             


                         70        80        90       100       110       120
                 =========+=========+=========+=========+=========+=========+
Ec-19_004670     --------------MAARVSTEAFCRAVVARVTVRDPKVGGTLKTSCSWELLEP---SAS
MSTRG.47812.1    KDGVSRDGVGVLNPMSARVSAEAFCRTLLARIAIRDPRVRSAIKPSCSWQLLKPPEPSSS
                               ########################################   ###


                        130       140       150       160       170       180
                 =========+=========+=========+=========+=========+=========+
Ec-19_004670     PVPVGKASRE---NKSTASIRRQLRSAQSLGLAGTRDDGAERLATQRSEEIVKRCGIHG-
MSTRG.47812.1    TAKNGNNSTQPDRHSHNTSICRQLHLAQSLGLAGDYDDGVLRGSSDRVEEIVKRCRMHGV
                 ########          ######################################### 


                        190       200       210       220       230       240
                 =========+=========+=========+=========+=========+=========+
Ec-19_004670     --------------GGGGGGG-----------TGGGTTDHDGKDFPSET-----------
MSTRG.47812.1    LYVDQDFPPKDSSLGPSLAASGLVTWSRSAQYPGAATTAFGGYGPPRPSDVRPCVFAMDA
                                                  #############              


                        250       260       270       280       290       300
                 =========+=========+=========+=========+=========+=========+
Ec-19_004670     -------------------------------AVANGRTD---ENRNG---------RGDH
MSTRG.47812.1    SLACALAALAERPHLISAALCGRVEEVVPLAAMSGGIQDGAWSSRAGSGGDCSEGLRSES
                                                                         ####


                        310       320       330       340       350       360
                 =========+=========+=========+=========+=========+=========+
Ec-19_004670     LALRKRTG--------------VATAEAAAKAFMAATESGIFCARLCADGVWKEYVLDDF
MSTRG.47812.1    CSPRRRSSGSENRKEGRKSSKRCIATAAAAHASTAATQAGMFSARLCVDGIWEEYVLDDF
                 ######                     #################################


                        370       380       390       400       410       420
                 =========+=========+=========+=========+=========+=========+
Ec-19_004670     FPCLSSYPERGGGSD--------------SGGGGPCLSRAHGPALWVSMLEKAYARVVGS
MSTRG.47812.1    FPCLSSNSDGASGGSRRRSGGRRCSGRGVSEGRGPCLSRAHGRAMWVSMLEKAYARAVGS
                 #############                ###############################


                        430       440       450       460       470       480
                 =========+=========+=========+=========+=========+=========+
Ec-19_004670     YSAALGGCCLP------CGPAKEREETEARAAASVVARPAKVLGVLTGSPVLQVDLSAGR
MSTRG.47812.1    YSDALGGCCMPPWGEGLEGTAVGPGVAATTSAAGTVARPAKLLGVFTGAPVLQVDLSETW
                 ###########                    #############################


                        490       500       510       520       530       540
                 =========+=========+=========+=========+=========+=========+
Ec-19_004670     RRAKDGEGVGVR----EDEDEEVAGELWGSIVSWARQGWLVCLSTPS--AETPEGGVHQE
MSTRG.47812.1    GKAGEEQGRSLGGSGYEADGDQATNELWESLVSWMRQGWLVCLSTAAPVKKEPEDARSQS
                 ########        #############################       ########


                        550       560       570       580       590       600
                 =========+=========+=========+=========+=========+=========+
Ec-19_004670     SGES------WKDGLRLGYAYTVLQTASVGSGHRLVRLRGPLLPKEGWRGKWTDDSPLWT
MSTRG.47812.1    GGGVGVAKGWWKDGLRLDHAYTVLQTADAGNGHRLVQVRGPLPAKEGWRGEWAEDSPLWA
                 ##        ################################################# 


                        610       620       630       640       650       660
                 =========+=========+=========+=========+=========+=========+
Ec-19_004670     ---------------QEALIAAQNSCPTHP---G------MSSYR--LEEPGAFWVSIEE
MSTRG.47812.1    MVPKVLMSVRAATAEADAAASQKNFLSTAAGDPDEERQQRRSSTRGDTEDGGAFWVSIDE
                                  ###########                    ############


                        670       680       690       700       710       720
                 =========+=========+=========+=========+=========+=========+
Ec-19_004670     AVSAFVEVGVCMVPLPPRTGG----DCRGHERILGGSRWA----HEVRRRVVFKRRRDRA
MSTRG.47812.1    AVSAFVEVGVCMTPPRPSTASGLLIGGRNHQQPQRKFGWCGVPTQESRRRVVFTRRGEDS
                 ###################        ############      ###############


                        730       740       750       760       770       780
                 =========+=========+=========+=========+=========+=========+
Ec-19_004670     GGGQGSGREYWGSGGGTAGIGAAQGKEEGGRGEEEEGGMLWGASQVYALSVYETSRIFFS
MSTRG.47812.1    GWQRGRGQSAPGTGGSEMPIGWKQ--QEHGCG-EEAGAVSWIPFQVYALSVYETSSMFFS
                 ########################         ###########################


                        790       800       810       820       830       840
                 =========+=========+=========+=========+=========+=========+
Ec-19_004670     VYQENRSDSASGVCWRDVGVTVLRVRDGCGPEVVASTGNPAQASVSTGTVLFPGRYVVIP
MSTRG.47812.1    LYQK-RGERGTERLYRDAGVTVLRVVDGRNPEVN--------------------------
                 ###            ##################                           


                        850       860       870       880       890       900
                 =========+=========+=========+=========+=========+=========+
Ec-19_004670     SYTGCVTKDQALQGNSELGNKNNHPVGVDNNDPLGKDEAPHQDSNSGHPLRTAESNPRET
MSTRG.47812.1    ------------------------------------------------------------
                                                                             


                        910       920       930       940       950       960
                 =========+=========+=========+=========+=========+=========+
Ec-19_004670     SNEKTWREGAEAKKGREGEEDSKKEAEVGLSFDQPEILKGLMTMFEGLDADCDGVLCREE
MSTRG.47812.1    ------------------------------------------------------------
                                                                             


                        970       980       990      1000      1010      1020
                 =========+=========+=========+=========+=========+=========+
Ec-19_004670     LDAFLRTTEGIPLQDDVYVWLVRSFSSCPRPSVLKDTHVSGHAETPRRVGAKGRRRPQRQ
MSTRG.47812.1    ------------------------------------------------------------
                                                                             


                       1030      1040      1050      1060      1070      1080
                 =========+=========+=========+=========+=========+=========+
Ec-19_004670     GRASRAPPAGIEDDGGCDGCHGGGGVHDVDLCLTRHGFVEMYRYIWQAAGRDFGVVMRDL
MSTRG.47812.1    ------------------------------------------------------------
                                                                             


                       1090      1100      1110      1120      1130      1140
                 =========+=========+=========+=========+=========+=========+
Ec-19_004670     RFAECHDARSEENRPSVVVAVHSDVQGFDFLAQPPDPELLEAAVALPIQRWGYPSPGYPD
MSTRG.47812.1    ------------------------------------------------------------
                                                                             


                       1150      1160      1170      1180      1190      1200
                 =========+=========+=========+=========+=========+=========+
Ec-19_004670     LASPPPVEHSRALATIASGSNKTGATTTAEMETATTASVTGHEITATFNNASRTDDDNEN
MSTRG.47812.1    ------------------------------------------------------------
                                                                             


                       1210      1220      1230      1240      1250      1260
                 =========+=========+=========+=========+=========+=========+
Ec-19_004670     ESNKARIIIDDAANNNNKSGLLLYVRPGPKGAENAVTFLVQSPIHFFSVRARLDCTGSVN
MSTRG.47812.1    ------------------------------------------------------------
                                                                             


                       1270      1280      1290      1300
                 =========+=========+=========+=========+========
Ec-19_004670     ASPMPGSIGLSREVTLLPGQSKVIQHLVPTDPRTPWSWKHAPLTVDTL
MSTRG.47812.1    ------------------------------------------------
```

```
Parameters used
Minimum Number Of Sequences For A Conserved Position: 2
Minimum Number Of Sequences For A Flanking Position: 2
Maximum Number Of Contiguous Nonconserved Positions: 8
Minimum Length Of A Block: 10
Allowed Gap Positions: None
Use Similarity Matrices: Yes
```

```
Flank positions of the 17 selected block(s)
Flanks: [75  114]  [118  128]  [139  179]  [214  226]  [297  306]  [328  373]  [390  431]  [452  488]  [497  525]  [533  542]  [551  599]  [618  628]  [649  679]  [688  699]  [706  744]  [754  783]  [796  813]  

New number of positions in Ec-19_004670.aa.fasta.trans.aa_ali.fasta-gb:  469  (35% of the original 1308 positions)
```
